# Significant but partial lipoprotein lipase functional loss caused by a novel occurrence of rare *LPL* biallelic variants

**DOI:** 10.1186/s12944-024-02086-0

**Published:** 2024-04-01

**Authors:** Yuepeng Hu, Jian-Min Chen, Han Zuo, Na Pu, Guofu Zhang, Yichen Duan, Gang Li, Zhihui Tong, Weiqin Li, Baiqiang Li, Qi Yang

**Affiliations:** 1 Department of Critical Care Medicine, Nanjing Jinling Hospital, Affiliated Hospital of Medical School, Nanjing University, Nanjing, China; 2 Univ Brest, Inserm, EFS, UMR 1078, GGB, Brest, F-29200 France; 3 Nanjing University of Chinese Medicine, Nanjing, China; 4 The First School of Clinical Medicine, Southern Medical University, Nanjing, China; 5 School of Basic Medical Sciences, Southern Medical University, Nanjing, China

**Keywords:** Biallelic variants, Compound heterozygote, Disease expression and severity, Familial chylomicronemia syndrome, Genotype/phenotype relationship, Hypertriglyceridemia-induced acute pancreatitis during pregnancy, Lipoprotein lipase, Partial loss-of-function variant, Triglyceride, Variant classification

## Abstract

**Background:**

Lipoprotein lipase (LPL) plays a crucial role in triglyceride hydrolysis. Rare biallelic variants in the *LPL* gene leading to complete or near-complete loss of function cause autosomal recessive familial chylomicronemia syndrome. However, rare biallelic *LPL* variants resulting in significant but partial loss of function are rarely documented. This study reports a novel occurrence of such rare biallelic *LPL* variants in a Chinese patient with hypertriglyceridemia-induced acute pancreatitis (HTG-AP) during pregnancy and provides an in-depth functional characterization.

**Methods:**

The complete coding sequences and adjacent intronic regions of the *LPL*, *APOC2*, *APOA5*, *LMF1*, and *GPIHBP1* genes were analyzed by Sanger sequencing. The aim was to identify rare variants, including nonsense, frameshift, missense, small in-frame deletions or insertions, and canonical splice site mutations. The functional impact of identified *LPL* missense variants on protein expression, secretion, and activity was assessed in HEK293T cells through single and co-transfection experiments, with and without heparin treatment.

**Results:**

Two rare *LPL* missense variants were identified in the patient: the previously reported c.809G > A (p.Arg270His) and a novel c.331G > C (p.Val111Leu). Genetic testing confirmed these variants were inherited biallelically. Functional analysis showed that the p.Arg270His variant resulted in a near-complete loss of LPL function due to effects on protein synthesis/stability, secretion, and enzymatic activity. In contrast, the p.Val111Leu variant retained approximately 32.3% of wild-type activity, without impacting protein synthesis, stability, or secretion. Co-transfection experiments indicated a combined activity level of 20.7%, suggesting no dominant negative interaction between the variants. The patient’s post-heparin plasma LPL activity was about 35% of control levels.

**Conclusions:**

This study presents a novel case of partial but significant loss-of-function biallelic *LPL* variants in a patient with HTG-AP during pregnancy. Our findings enhance the understanding of the nuanced relationship between *LPL* genotypes and clinical phenotypes, highlighting the importance of residual LPL function in disease manifestation and severity. Additionally, our study underscores the challenges in classifying partial loss-of-function variants in classical Mendelian disease genes according to the American College of Medical Genetics and Genomics (ACMG)’s variant classification guidelines.

## Background

The lipoprotein lipase (*LPL*) gene is located on chromosome 8p21.3 and comprises 10 exons, encoding a precursor protein of 475 amino acids. The mature LPL protein, consisting of 448 amino acids, plays a crucial role as the central enzyme in the regulation of triglyceride (TG) hydrolysis [1, 2]. *LPL* stands out as one of the most extensively studied human disease genes, with investigations spanning from a Mendelian disorder recognized as autosomal recessive familial chylomicronemia syndrome (FCS), also known as type I hyperlipoproteinemia or LPL deficiency [3], to a complex trait, hypertriglyceridemia (HTG) [4, 5], and its associated conditions, including HTG-induced acute pancreatitis (HTG-AP) [6].

FCS is a rare genetic disorder with an estimated prevalence of 1–2 individuals per million [7]. It represents a severe condition that disrupts the proper breakdown of dietary fats, resulting in an accumulation of TGs. The hallmark manifestation of FCS is the substantial elevation of plasma TG levels (> 10 mmol/L or 880 mg/dL), typically emerging during infancy or early childhood. Additional FCS symptoms include failure to thrive, abdominal pain, nausea, vomiting, and potential progression to acute pancreatitis (AP). Further manifestations encompass fatigue, irritability, lipemia retinalis, eruptive xanthomas on the trunk, back, and gluteal region, along with hepatosplenomegaly [8]. The primary genetic cause of FCS (accounting for 80–90% of cases) is attributed to complete or near-complete loss of LPL function due to rare biallelic (homozygous or compound heterozygous) *LPL* variants [8].

Rare biallelic *LPL* variants have also been previously reported in three patients with HTG-AP during pregnancy [9, 10]. Pregnancy, a physiological state that is normally associated with a 2- to 4-fold increase in serum TG levels in late gestation [11], may be regarded as a unique type of environmental factor. While most pregnant women with normal baseline TG levels can tolerate this TG increase, those with genetic defects in TG metabolism genes are prone to severe HTG and, consequently, HTG-AP. Thus, in essence, HTG-AP during pregnancy is a complex condition arising from the interplay between genetic risk factors for HTG and the unique metabolic demands of pregnancy [12].

Returning to the three previously reported patients with HTG-AP during pregnancy, none had a prior history of HTG or AP [9, 10]. One patient was homozygous for c.596 C > G (p.Ser199Cys), while another was compound heterozygous for c.836T > G (p.Leu279Arg) and c.862G > A (p.Ala288Thr), and the third patient was compound heterozygous for c.805G > A (p.Glu269Lys) and c.835 C > G (p.Leu279Val). Notably, all these patients exhibited a significant level of plasma LPL activity, ranging from 12 to 25% of controls. Consistent with this, functional analysis revealed that the p.Ser199Cys and p.Ala288Thr variants caused a significant but partial loss of LPL function. To the best of our knowledge, this was the first study that identified specific partial loss-of-function (LoF) rare biallelic *LPL* variants associated with milder phenotypic manifestations compared to classic FCS. These findings substantially advanced our understanding of the intricate relationship between *LPL* genotypes and clinical phenotypes, highlighting the crucial role of residual LPL function in disease expression and severity. In our current study, we expand upon these prior discoveries by presenting a novel occurrence of rare biallelic *LPL* variants, which encompass a known missense variant and a novel missense variant, in a Chinese patient diagnosed with HTG-AP during pregnancy.

## Methods

### Ethics statement

This study received approval from the Ethics Committee of Jinling Hospital in Nanjing (2021NZKY-042-01), China. Informed consent was obtained from each participant.

### Patient description

The patient under investigation was a 30-year-old pregnant woman of Chinese ethnicity who was admitted to a local hospital at 38^+ 2^ weeks of gestation due to sudden-onset abdominal pain, ultimately undergoing an emergency cesarean section. Upon admission, her serum amylase level was measured at 800 U/L, and her blood sample exhibited chylous characteristics. In conjunction with pancreas imaging findings, she received a diagnosis of HTG-AP during pregnancy. Following a 20-day treatment regimen at the local hospital, she was subsequently transferred to our facility at Jinling Hospital for further treatment, with her TG level measured at 2.8 mmol/L. After a total of 98 days from her initial admission, she was discharged from our hospital. Her TG levels have consistently remained well-controlled, maintained at approximately ∼ 3 mmol/L through her daily fenofibrate medication.

Prior to her pregnancy, the patient had a six-year history of HTG, with her TG level measured at ∼ 5 mmol/L at the time of diagnosis. She had consistently received treatment with fenofibrate at a daily dosage of 100 mg since her HTG diagnosis, effectively maintaining her TG levels at ∼ 3 mmol/L before her pregnancy. The patient had not reported any episodes of AP or other significant medical conditions. She did not smoke or consume alcohol and maintained a normal body weight before becoming pregnant.

The patient’s father had a known history of HTG (TG level, ∼ 4 mmol/L), while the brother, son, and deceased mother did not exhibit this condition.

### Genetic analysis

In accordance with our previous publications [13, 14], our genetic analysis of the patient involved the following steps: (i) Performing Sanger sequencing to cover the complete coding sequences and adjacent intronic regions of the *LPL* gene, along with four other key genes related to TG regulation, namely, *APOC2*, *APOA5*, *LMF1*, and *GPIHBP1*; (ii) Focusing on rare variants, defined as those with an allele frequency of < 0.01 using global population data from the Genome Aggregation Database (gnomAD) [15]); and (iii) Including nonsense, frameshift, missense, small in-frame deletions or insertions, and canonical GT-AG splice site variants for subsequent analysis.

The reference *LPL* mRNA sequence utilized in this study was NM_000237.3. *LPL* variants were designated following the guidelines established by the Human Genome Variation Society (HGVS) [16].

### *In silico* analyses

We evaluated the evolutionary conservation of LPL positions p.Val111 and p.Arg270, following the methodology described in our previous work [17]. This assessment involved utilizing a multiple-species protein sequence alignment and various tools, including Genomic Evolutionary Rate Profiling (GERP; http://mendel.stanford.edu/sidowlab/downloads/gerp/index.html), phastCons46way (https://genome.ucsc.edu/cgi-bin/hgTrackUi?db=hg19&g=cons46way), phastCons100way (https://genome.ucsc.edu/cgi-bin/hgTrackUi?db=hg19&g=cons100way), phyloP46way ((https://hgdownload.soe.ucsc.edu/goldenPath/hg19/phyloP46way/), and phyloP100way (http://hgdownload.soe.ucsc.edu/goldenPath/hg38/phyloP100way/).

We also employed four prediction tools—SIFT (https://sift-ag.com/), PolyPhen2_HDIV (http://genetics.bwh.harvard.edu/pph2/), PolyPhen2_HVAR (http://genetics.bwh.harvard.edu/pph2/), and PROVEAN (https://www.jcvi.org/research/provean)—to assess the pathogenicity of the *LPL* p.Val111Leu and p.Arg270His missense variants. Additionally, we used PyMOL software (https://pymol.org/2/) to predict the 3D structures of both wild-type (WT) and variant LPL proteins.

### Plasmid construction, cell culture, and transfection

The following procedures were carried out in accordance with previously established protocols [17]. Specifically, human WT, c.331G > C, and c.809G > A *LPL* cDNAs were synthesized and subsequently cloned into the pcDNA3.1 vector by GenScript (GenScript Biotech Corporation, Nanjing, China). Plasmid constructions were validated through Sanger sequencing.

The HEK293T cell line (ATCC, CRL-3216), which lacks endogenous LPL expression, was cultured in DMEM supplemented with 10% FBS and 1% penicillin-streptomycin (PS) at 37 °C in a humidified incubator with 5% CO_2_. Transient transfections were conducted for a duration of six hours in 6-well plates (Corning, 354,573) using a 2 ml volume of medium.

For single plasmid transfection, the expression vector was at a concentration of 1500 ng/ml, while for co-transfection of two plasmids, the concentration of each expression vector was 750 ng/ml. The transfection procedure was carried out using Lipofectamine 3000 (Thermo, L3000015) in accordance with the manufacturer’s instructions.

Following transfection, the cells were cultured for an additional 48 h in DMEM supplemented with 2% FBS and 1% PS before being harvested for the quantification of expressed LPL proteins. In experiments involving LPL secretion and catalytic activity, the transfected cells were further cultured for 30 min in 500 µl of DEME medium containing 100 U/L heparin. Subsequently, both the cells and the medium were collected for LPL mass and/or activity analysis.

### Analysis of LPL mass and activity in transfected cells and medium

For experiments involving transfected cell medium, the cell medium underwent centrifugation at 4 °C for 10 min at 12,000 rpm to remove cells and debris. Subsequently, the resulting supernatant was meticulously collected and then stored at -80 °C for subsequent analysis. For experiments with transfected cells, the transfected cells were first harvested and treated with RIPA Lysis Buffer (Beyotime, China, P0013) for a duration of 30 min. After this treatment, the cell suspension was subjected to centrifugation at 4 °C for 10 min at 12,000 rpm. The resulting supernatants were collected with care and likewise stored at -80 °C for further analysis.

Western blotting was employed to assess the expression of LPL protein in both the cell medium and lysate, as previously outlined [17]. The antibodies and their respective dilutions utilized in this study were as follows: primary rabbit LPL antibody (Santa Cruz Biotechnology, 73,646) at a 1:200 dilution, primary mouse GAPDH antibody (Santa Cruz Biotechnology, 47,724) at a 1:5000 dilution, secondary anti-rabbit IgG-HRP (Santa Cruz Biotechnology, 2357) at a 1:2000 dilution, and secondary anti-mouse IgG-HRP (Santa Cruz Biotechnology, 2004) at a 1:5000 dilution. The protein bands were visualized and quantified using the Chemidoc XRS System, Image Lab Software (Clinx Science Instruments, Shanghai, China), with normalization to GAPDH.

The analysis of LPL activity in the cell medium was conducted following the below described protocol used for post-heparin plasma.

All Western blot assays were conducted independently, with each experiment being replicated twice, incorporating two samples for each replicate. To evaluate the relative expression levels of the mutant LPL protein, we calculated the ratio of grayscale intensities between the LPL protein bands and the β-actin protein bands. This quantitative assessment of the mutant LPL protein expression was then expressed as a percentage relative to the ratio observed in the wild-type control. The enzyme activity assays were conducted with three independent replications, each comprising three samples. Results from these assays were similarly presented as percentages. Data were expressed as mean ± standard deviation (SD) and analyzed using GraphPad Prism 6.01 (GraphPad Software, Inc.) for graphical presentations and SPSS 25.0 (IBM Analytics, Armonk, NY) for statistical analysis. A *P*-value of less than 0.05 was considered statistically significant.

### Analysis of LPL activity in post-heparin plasma

This assay was performed following established methods as described previously [18]. Briefly, a blood sample was collected from the patient following an overnight fast and 10 min after the intravenous administration of heparin (60 IU/kg of body weight). Blood plasma was prepared by centrifugation at 400 g for 30 min. To assess LPL activity, we initially quantified plasma total lipase activity and hepatic lipase activity through an LPL-mediated lipolysis reaction. This was carried out using a free fatty acid (FFA) release assay kit [Wako kit# NEFA-HR(2), Japan], with TG-rich plasma obtained from *Gpihbp11*-deficient (*Gpihbp11*^*–/–*^) mice as the substrate for lipolysis. In the case of the plasma hepatic lipase activity assay, the sample was pretreated with 1 M NaCl and incubated for 60 min at 4 °C to inactivate the LPL. Subtracting hepatic lipase activity from total lipase activity provided a measure of plasma LPL activity. Both assays were conducted with three technical replicates. The reference value for normal LPL activity was derived from data obtained from 10 healthy volunteers at our center, consisting of 5 males and 5 females with an average age of 28.8 years.

## Results

### Discovery of a novel instance of rare biallelic *LPL* variants in the patient

**Fig. 1:**
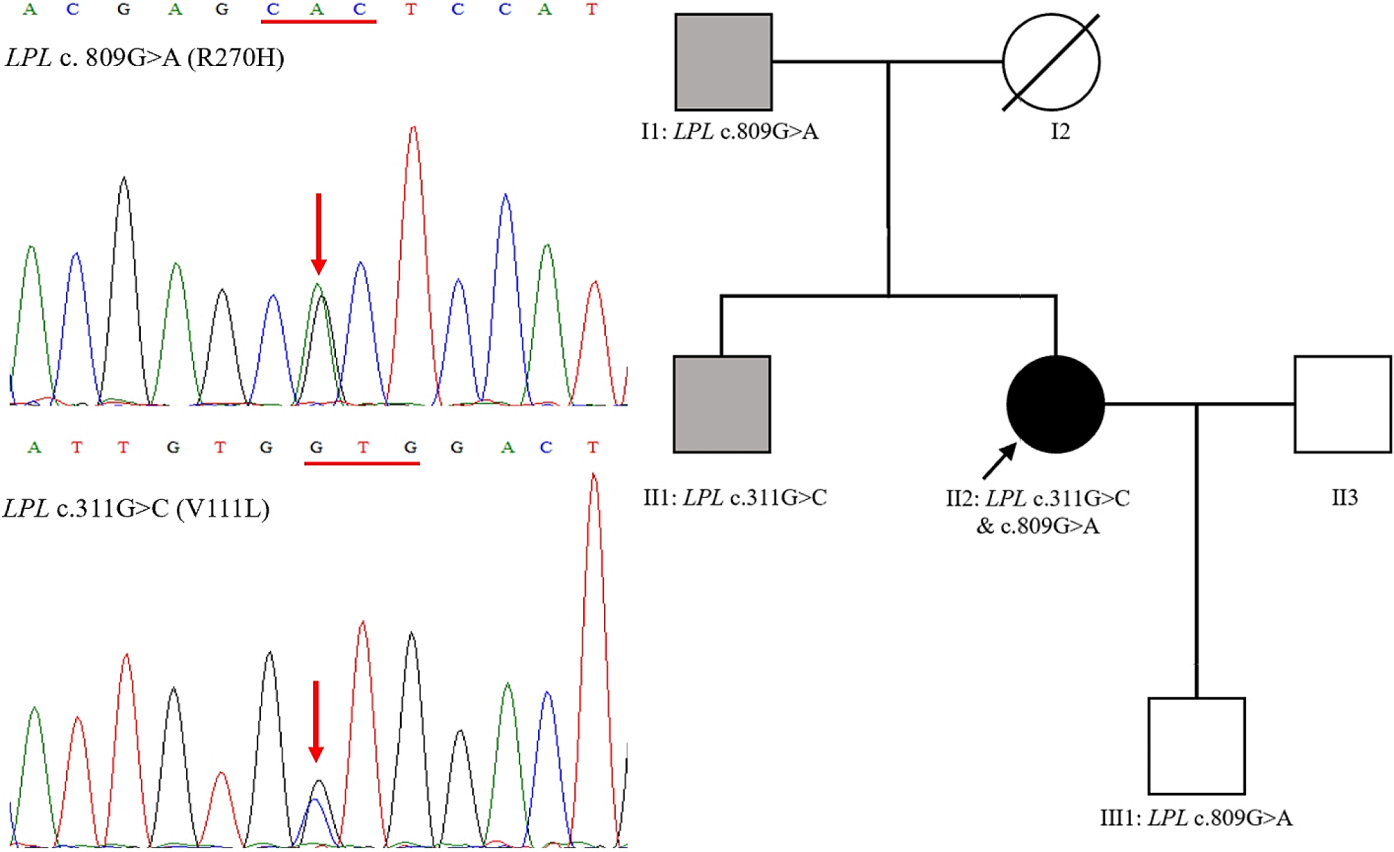
Discovery of rare biallelic *LPL* variants in a patient with HTG-AP during pregnancy. Left panel, sequencing electropherogram displaying the rare biallelic *LPL* variants, p.Val111Leu and p.Arg270His. Right panel, family pedigree of the proband with HTG-AP during pregnancy. The proband is denoted by an arrow. Grey shading represents individuals with HTG. *LPL* variant(s) are labeled below the individuals who underwent genetic analysis. *Abbreviations* HTG, hypertriglyceridemia; HTG-AP, HTG-induced acute pancreatitis

In our analysis, we identified only two variants that met our inclusion criteria in the patient. Both of these variants were located within the *LPL* gene and classified as missense variants. Specifically, these variants were c.331G > C (p.Val111Leu) and c.809G > A (p.Arg270His) (Fig. 1, left panel), situated in exons 3 and 6 of the *LPL* gene, respectively. Notably, c.331G > C was not found in gnomAD v4.0.0 [15], while c.809G > A had a global population allele frequency of 0.00001239.

Additionally, we conducted genetic analyses on the patient’s father, brother, and son to investigate these genetic variations. The father and son were found to carry the p.Arg270His variant, while the brother harbored the p.Val111Leu variant (Fig. 1, right panel). These findings confirm that the two variants identified in the patient were inherited from the father and mother, respectively, establishing the patient as a compound heterozygote for the two *LPL* missense variants.

Furthermore, p.Arg270His had been previously reported in the literature [19–23] and is documented in the ClinVar database [24]. This variant was consistently identified in patients with classical FCS, whether in a homozygous or compound heterozygous state [19–23]. Notably, the first three reports conducted in vitro functional characterizations of the p.Arg270His variant and consistently found no LPL activity in the medium of the transfected cells [19–21]. However, p.Val111Leu represents a novel variant, signifying the identification of a unique instance of rare biallelic *LPL* variants in the patient.

### *In silico* analyses of the two *LPL* missense variants

**Fig. 2:**
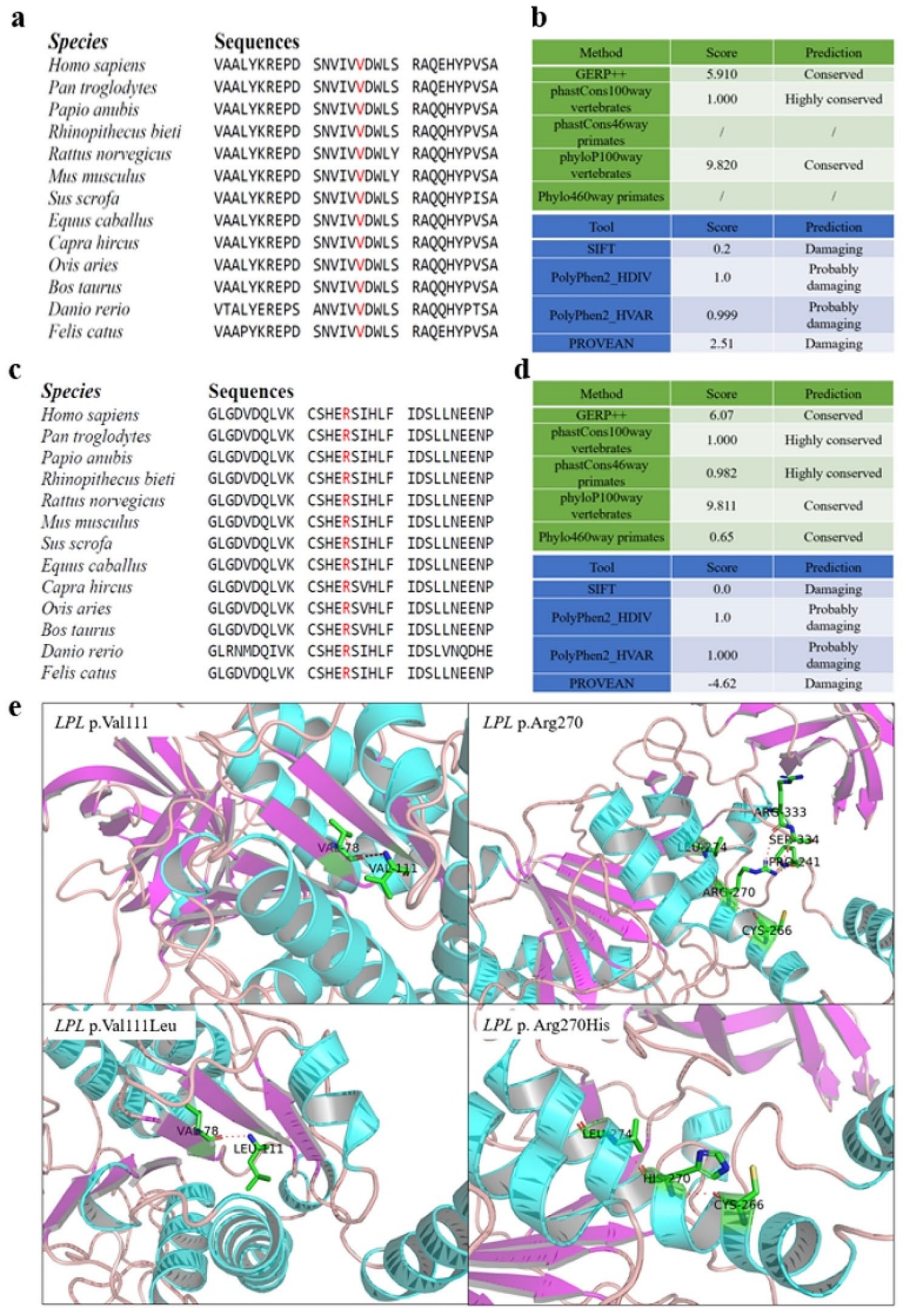
*In silico* analyses pertaining to the *LPL* p.Val111Leu and p.Arg270His variants. (**a**) Alignment of partial vertebrate LPL amino acid sequences spanning the p.Val111 site. (**b**) Conservation and pathogenicity scores of the LPL p.Val111 site as predicted by the indicated programs. (**c**) Alignment of partial vertebrate LPL amino acid sequences spanning the p.Arg270 site. (**d**) Conservation and pathogenicity scores of the LPL p.Arg270 site as predicted by the indicated programs. (**e**) Predicted partial 3D structures of the wild-type and variant LPL proteins

Next, we compared the potential functional impact of p.Val111Leu with that of p.Arg270His at the protein level, revealing a high degree of similarity in all three assessed aspects (Fig. 2). Specifically, both the p.Val111 and p.Arg270 positions exhibited evolutionary conservation, and both the p.Val111Leu and p.Arg270His missense variants were predicted to be damaging or highly deleterious. Furthermore, both missense variants were predicted to significantly impact the protein’s 3D structure. These predictions strongly suggest that, similar to p.Arg270His, p.Val111Leu may also significantly affect LPL function.

### Functional characterization of the two *LPL* missense variants in transfected cells and medium

Then, we conducted a functional characterization of the p.Val111Leu variant in transfected HEK293T cells and medium, using the p.Arg270His variant and WT *LPL* sequences as controls.

**Fig. 3:**
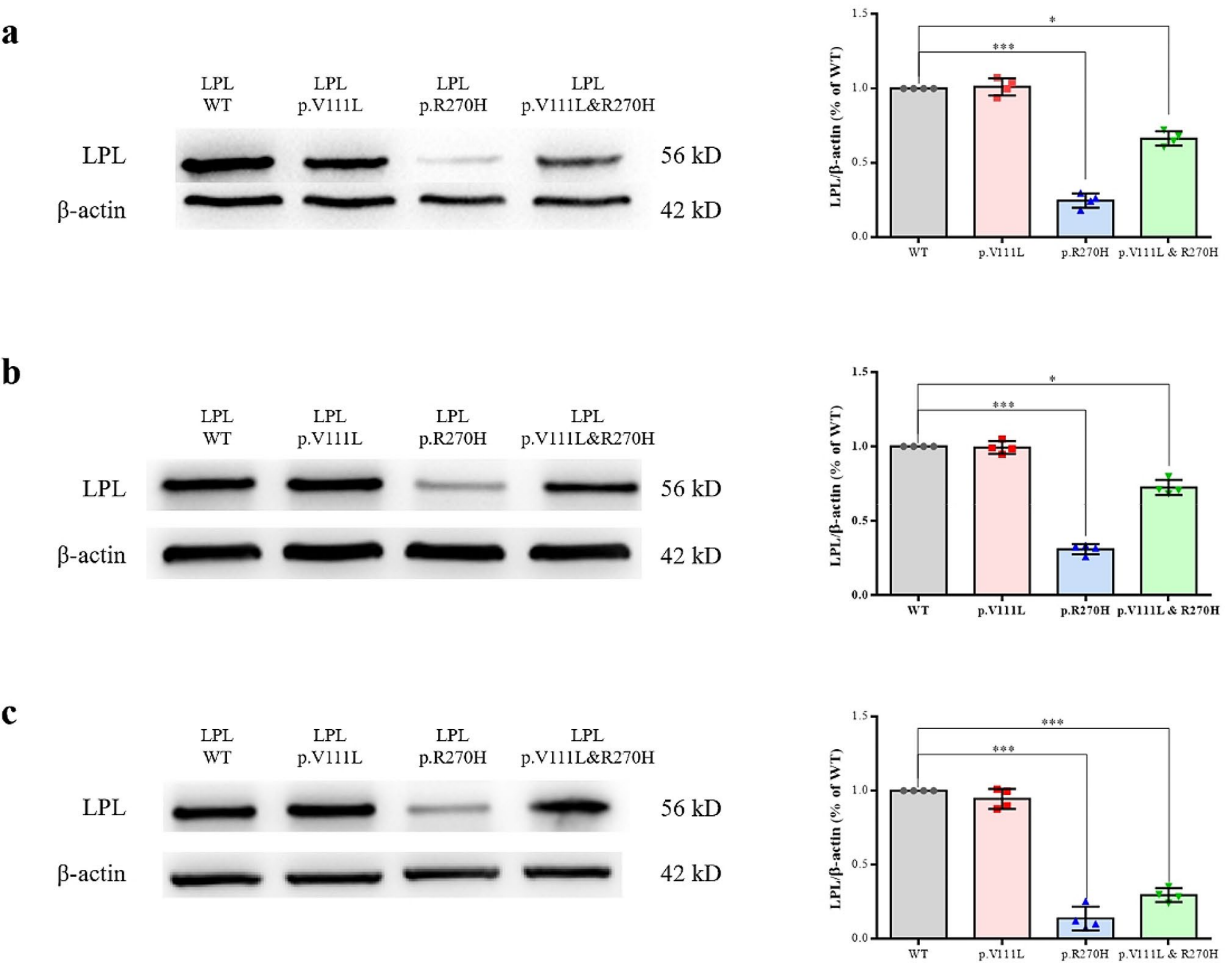
Functional characterization of missense variants *LPL* p.Val111Leu and p.Arg270His. Western blot analyses assessed LPL expression in HEK293T cell lysates or medium following transfection with wild-type (WT) or variant *LPL* constructs, under conditions with and without heparin treatment. (**a**) LPL expression in lysates from cells without heparin treatment. (**b**) LPL expression in the medium after heparin treatment. (**c**) LPL expression in lysates from cells with heparin treatment. In each panel, the left subpanel shows a representative blot, while the right subpanel provides quantification of LPL expression, with band intensities normalized to β-actin. ‘p.V111L & p.R270H’ denotes co-transfection with both variant constructs. *, *P* < 0.05; ***, *P* < 0.001

In the initial phase, we conducted single-plasmid transfection experiments, both with and without heparin treatment (heparin was used to stimulate LPL secretion). To assess the impact on LPL synthesis/stability, we analyzed LPL mass data from three sources: (i) lysate of transfected cells without heparin treatment, (ii) lysate of transfected cells with heparin treatment, and (iii) medium of transfected cells with heparin treatment (Fig. 3). Our analysis revealed that the p.Arg270His variant primarily affects LPL synthesis/stability, with the p.Arg270His mutant mass in the lysate of transfected HEK293T cells representing only ∼ 25% of the WT. In contrast, the novel p.Val111Leu variant showed no appreciable effect on LPL synthesis/stability and secretion (Fig. 3).

**Fig. 4:**
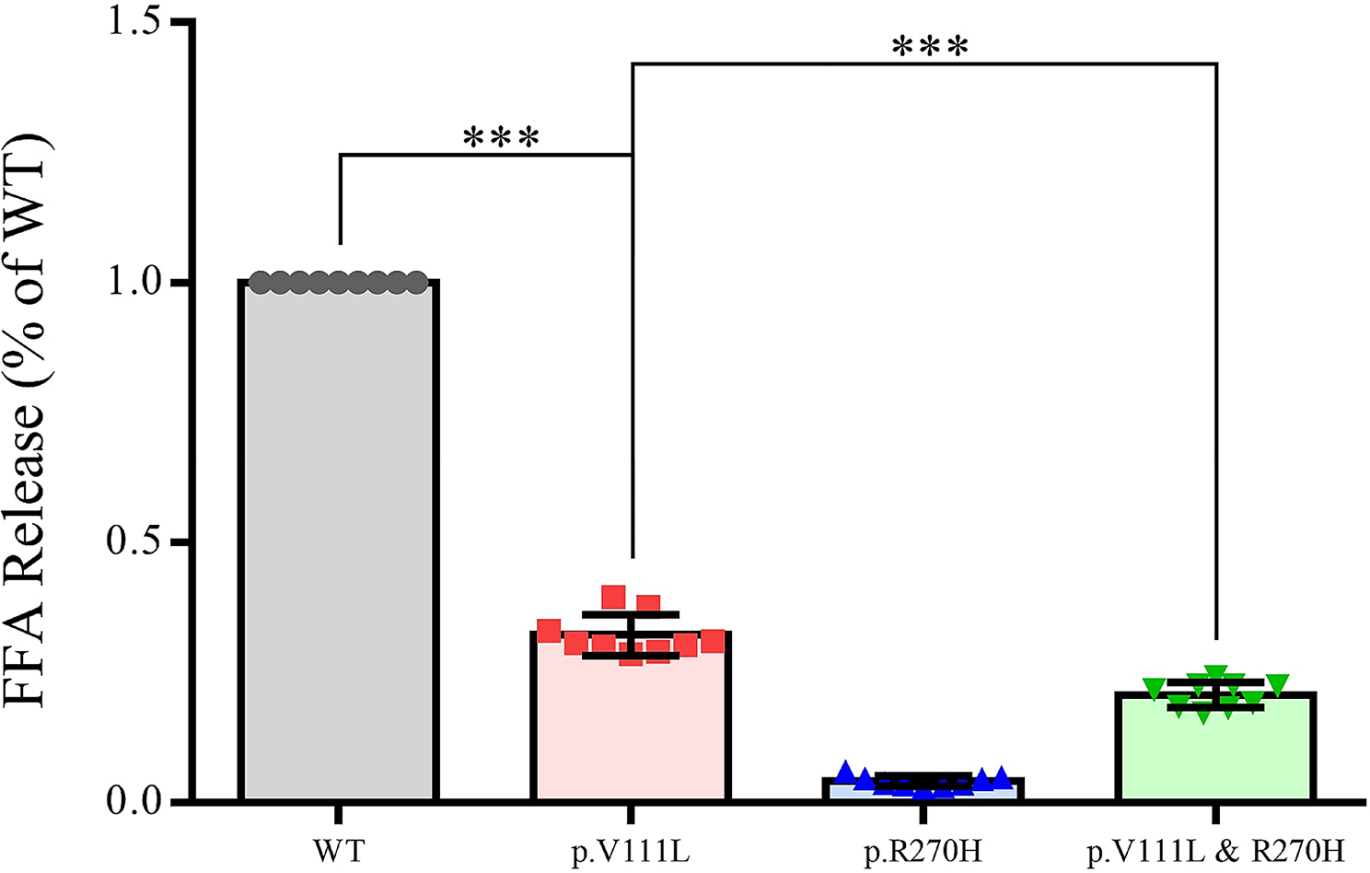
Relative activity of LPL in the culture medium of cells transfected with either wild-type (WT) or variant LPL constructs, following heparin treatment. The activity level of the WT construct is set as a baseline value of 1. Data are presented as mean ± standard deviation (SD) derived from three independent transfections, with each assay conducted in triplicate. ***, *P* < 0.001. *Abbreviations* FFA, free fatty acid

Furthermore, the LPL activity in the medium of HEK293T cells transfected with the p.Arg270His expression vector was barely detectable (Fig. 4), consistent with the findings from all three previous publications [19–21]. In contrast, for the novel p.Val111Leu variant, the corresponding value was 32.3% of WT (Fig. 4).

In summary, our initial set of functional analyses, conducted through single-plasmid transfections, revealed that the novel p.Val111Leu variant had a modest impact on LPL activity. More specifically, it retains approximately 32.3% of WT activity while not affecting LPL synthesis, stability, or secretion. In contrast, the p.Arg270His variant had a profound impact, leading to an almost complete loss of LPL function. This loss of function was attributed to a combined effect on protein synthesis/stability, secretion, and enzyme activity.

In the subsequent phase, we conducted co-transfection experiments due to the compound heterozygous nature of the two missense variants in the patient. Both LPL mass and activity assays revealed an additive combined effect of the two missense variants (Figs. 3 and 4). For instance, the LPL activity in the medium of HEK293 cells co-transfected with both missense variants (i.e., 20.7% of WT) is approximately half the sum of the LPL activities from the two separate transfection studies (i.e., 36.4% of WT). These results effectively rule out any dominant negative effect between the two missense variants.

### Post-heparin plasma LPL activity in the patient

Finally, we measured the patient’s post-heparin plasma LPL activity. This measurement occurred 189 days after her initial admission to the local hospital. The patient’s post-heparin plasma LPL activity was determined to be ∼ 35% of the normal LPL activity, which was derived from a control group of 10 healthy volunteers at our center, comprising five males and five females with an average age of 28.8 years.

For comparison, we have compiled clinical and functional analytical data for our patient alongside information on the three previously reported patients with HTG-AP during pregnancy in Table 1.

**Table 1:** Summary of key clinical, genetic, and functional analysis data for rare biallelic *LPL* variants in patients with HTG-AP during pregnancy

Biallelic variants	Zygosity	Plasma LPL activity (% of controls)	Age at genetic analysis	Disease history	In vitro LPL activity (% of wild-type)	Reference
c.596 C > G (p.Ser199Cys)	Homozygote	12	30 years	Developed severe chylomicronemia and pancreatitis for the first time at 33 weeks of gestation at age 30; had no history of HTG	6	[9]
c.836T > G (p.Leu279Arg) c.862G > A (p.Ala288Thr)	Compound heterozygote	25	37 years	Developed severe pancreatitis with a plasma TG level of 162 mmol/L at 29 weeks of gestation during her first pregnancy; her blood glucose, liver, thyroid, and renal functions were found to be normal; had no personal or family history of hyperlipidemia or pancreatitis	p.Leu279Arg: 0 p.Ala288Thr: 32–36 Co-transfection: 20	[9]
c.805G > A (p.Glu269Lys) c.835 C > G (p.Leu279Val)	Compound heterozygote	17	30 years	Hospitalized for severe abdominal pain, vomiting, and nausea during the 20th week of pregnancy; no prior history of significant medical conditions.	Not available	[10]
c.331G > C (p.Val111Leu) c.809G > A (p.Arg270His)	Compound heterozygote	35	30 years	HTG-AP at 38^+ 2^ weeks of gestation; had a 6-year history of HTG but no AP history or other diseases	p.Val111Leu: 32.3 p.Arg270His: 4.1 Co-transfection: 20.7	This study

*Abbreviations* AP, acute pancreatitis; HTG, hypertriglyceridemia; HTG-AP, hypertriglyceridemia-induced acute pancreatitis; LPL, lipoprotein lipase; TG, triglyceride

## Discussion

As illustrated in Table 1, our case exhibits remarkable similarities to the three previously reported cases in terms of clinical, genetic, and functional analytical data. These cases shared milder phenotypic manifestations, compared to classic FCS, which were attributed to the retention of a significant level of plasma LPL activity, stemming from their partial LoF *LPL* genotypes. These findings may serve as a catalyst for new studies focusing on rare biallelic *LPL* variants. The accumulation of more data may ultimately lead to the identification of a threshold of residual LPL function that safeguards against classical FCS, potentially offering valuable insights into tailored therapeutic approaches for affected individuals.

Among the seven missense variants identified in the four patients (Table 1), five underwent functional characterization. Of these, two variants, namely p.Leu279Arg and p.Arg270His, caused complete or nearly complete functional loss. Following the variant classification guidelines provided by the American College of Medical Genetics and Genomics (ACMG) [25], these two variants can be confidently categorized as “pathogenic”, each supported by two strong pieces of evidence (PS3 and PS4). However, a challenge arises when considering the classification of the other three partial LoF missense variants, namely p.Ser199Cys, p.Ala288Thr, and p.Val111Leu. It seems inappropriate to categorize them as “pathogenic” since none of them alone would lead to FCS when inherited with a complete LoF *LPL* variant on the other chromosome. Furthermore, designating them as “variants of uncertain significance” appears inadequate, as they are clearly disease-associated and significantly affect protein function [26]. Drawing from the *LDLR*-specific ACMG guidelines [27], implementing a threshold of functional loss could offer a practical solution for classifying these variants. However, it is crucial to note that the optimal approach to classify partial LoF variants is still a matter of active discussion within the scientific community, as evident from recent debates and scholarly articles [28, 29]. This ongoing conversation highlights the intricacies and dynamic nature of genetic variant classification, especially regarding their implications in disease contexts.

### Study strengths and limitations

The strengths of our study include (i) providing comprehensive clinical and family data of the proband, and (ii) undertaking a rigorous functional analysis of the two identified *LPL* missense variants. A common limitation shared by our study and others in the field is the reliance on a single cellular model (HEK293T cells) for functional analyses. While this model is widely used for its ease of transfection and relevance in studying protein expression and function, it may not fully reflect in vivo situations.

## Conclusions

In this study, we have presented a novel case involving partial LoF rare biallelic *LPL* variants in a patient with HTG-AP during pregnancy. Our findings contribute to the growing body of evidence linking partial LoF *LPL* variants to non-FCS phenotypes and emphasize the challenges in applying current ACMG guidelines to classify such variants.

## Data Availability

All supporting data are available within the article

## Abbreviations

ACMG: the American College of Medical Genetics and Genomics
AP: acute pancreatitis
FCS: familial chylomicronemia syndrome
gnomAD: the Genome Aggregation Database
HTG: hypertriglyceridemia
HTG-AP: hypertriglyceridemia-related acute pancreatitis
LoF: loss-of-function
LPL: lipoprotein lipase
TG: triglyceride,WT,wild-type
